# Food Decision-Making: Effects of Weight Status and Age

**DOI:** 10.1007/s11892-016-0773-z

**Published:** 2016-07-29

**Authors:** Floor van Meer, Lisette Charbonnier, Paul A. M. Smeets

**Affiliations:** 1 Image Sciences Institute, Brain Center Rudolf Magnus, University Medical Center Utrecht, room Q02.445, Heidelberglaan 100, 3584 CX Utrecht, The Netherlands; 2 Division of Human Nutrition, Wageningen University & Research Centre (Bode 62), 8129, 6700 EV Wageningen, The Netherlands

**Keywords:** Food choice, Decision-making, Obesity, Development, Neural correlates

## Abstract

Food decisions determine energy intake. Since overconsumption is the main driver of obesity, the effects of weight status on food decision-making are of increasing interest. An additional factor of interest is age, given the rise in childhood obesity, weight gain with aging, and the increased chance of type 2 diabetes in the elderly. The effects of weight status and age on food preference, food cue sensitivity, and self-control are discussed, as these are important components of food decision-making. Furthermore, the neural correlates of food anticipation and choice and how these are affected by weight status and age are discussed. Behavioral studies show that in particular, poor self-control may have an adverse effect on food choice in children and adults with overweight and obesity. Neuroimaging studies show that overweight and obese individuals have altered neural responses to food in brain areas related to reward, self-control, and interoception. Longitudinal studies across the lifespan will be invaluable to unravel the causal factors driving (changes in) food choice, overconsumption, and weight gain.

## Introduction

**Fig. 1:**
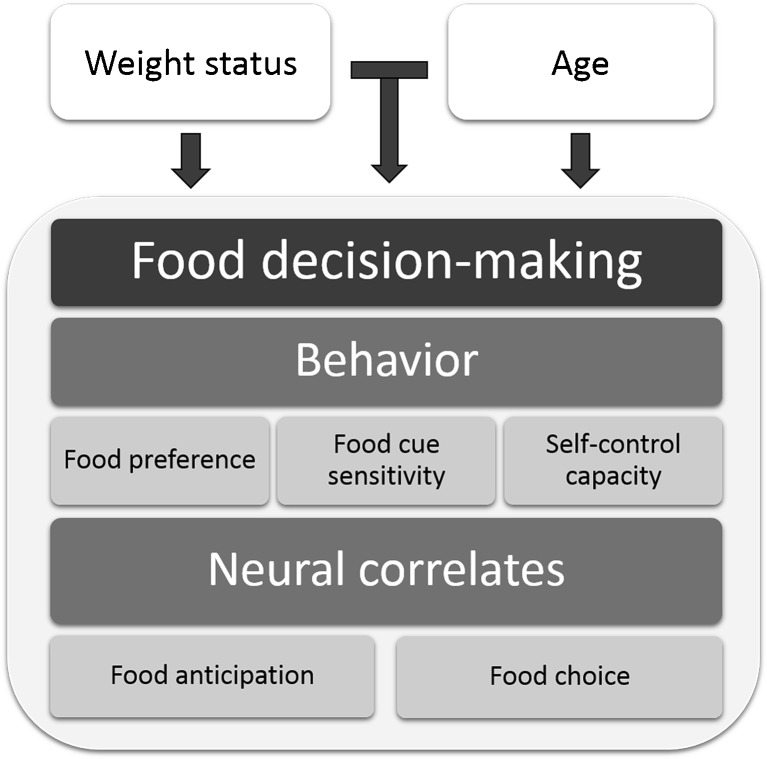
Schematic overview of the factors affecting food decision-making which are discussed in this review. Note that external factors are outside the scope. The effects of weight status and age on factors examined in behavioral studies (food preference, food cue sensitivity, and self-control capacity) and factors examined in neuroimaging studies (food anticipation and food choice) are shown in the order in which they are discussed in the text

People make over 200 food decisions per day [1]. Food decisions are the choices made concerning what, when, and how much to eat. Together, they determine energy and nutrient intake. When more energy is consumed than is expended, e.g., by eating energy-dense fast foods, overconsumption occurs. Since overconsumption causes a positive energy balance, which leads to weight gain, it is considered to be a main cause of obesity [2]. Rates of childhood obesity are rising at an alarming rate [3], and the chance that an obese child develops into an obese adult is much higher than that of a normal-weight child. Moreover, once people have become overweight or obese, it is quite challenging for them to revert to a stable healthy weight. Thus, prevention of overconsumption is crucial [4], and this requires knowledge on the drivers of food decision-making and how these are affected by weight status. Furthermore, since the prevalence of overweight, obesity, and type 2 diabetes increases with age, determinants of food decisions in older adults are of vital importance as well. Although food choices are affected by many factors, such as availability, cultural, economic, and ethical considerations, this review focuses on the effects of weight status and age, as two key characteristics. To give a comprehensive overview of how weight status and age influence food decision-making, both behavioral and neuroscience studies will be discussed (Fig. 1). We aim to provide an understanding of the causes of maladaptive food decisions and identify knowledge gaps and new avenues for possible interventions.

### Food Choice Behavior

There are many models of food choice, ranging from socio-psychological and cultural models to economic models. Food choice behavior has been studied with many research methodologies, such as qualitative measures, food frequency questionnaires, food choice tasks, intake measurements, eye-tracking, and measurements of purchase. We grouped the literature into three topics that are relevant for understanding the role of weight status and age in food choice behavior: food preference, food cue sensitivity, and self-control capacity (Fig. 1, Table 1).

**Table 1:** Key influencers of food decision-making and the strength of evidence for effects of weight status and age

	Weight status	Age
Food preference	±	+
Food cue sensitivity	±	+
Self-control capacity	+	+

+ denotes factor has been shown to have an effect. ± denotes evidence for this factor having an effect is conditional or inconclusive

#### Food Preference

Nutrient and energy rich foods appear to be naturally attractive to all humans [5]. Food preferences are largely learned by experience; only sweet taste preference is inborn [6]. It has been hypothesized that an innate preference for energy-dense foods leads to higher consumption of these foods and thus obesity [7]. However, overweight and obese individuals do not give higher preference ratings when tasting food (both high and low energy) than normal-weight individuals [8]. Furthermore, in both children and adults, there is no clear evidence for a relationship between taste sensitivity or preference for sweet, salty, sour or bitter tastes, and weight status [9•] (review in which studies that use taste sensitivity, taste preference, and hedonic preference measures were included). There is some evidence for a higher preference for fatty foods in overweight and obese individuals and higher preference for salty foods in overweight and obese children [9•]. Taken together, it is more likely that obesity is related to problems in dealing with food cues and the motivation to eat, than with heightened pleasure derived from eating or a stronger preference for energy-dense foods [7, 9•].

Children’s food preferences are highly determined by their experiences with the food and the preferences of their parents [10]. Repeatedly exposing children to foods, for example green vegetable soup, increases preference and consumption [11]. Unfortunately, children are often exposed to advertisements for unhealthy snack foods, e.g. on television, which increases their preference for foods high in fat, sugar, and salt [12]. Children’s initial preferences last throughout adolescence, but may change as they eat more meals outside their home [13]. Elderly people experience loss of appetite associated with aging and a functional decline of taste and smell that may lead to decreases in food palatability [14] and may change their food choices towards more intense flavors [15]. In conclusion, higher preference for fat may be linked to overweight, but evidence for this is marginal. Although food preferences change over the lifespan, there are no studies showing that this leads to a changed risk of overconsumption.

#### Food Cue Sensitivity

Food cues are relevant for everyone from a biological perspective. In line with this, hungry normal-weight individuals have an attentional bias towards food cues (using a dot probe task [16] with food-related words), since these are then more relevant [17]. When sated, normal-weight subjects have a diminished attentional bias towards food cues, while overweight women have been found to exhibit greater attention for food compared to non-food cues when satiated, as measured with eye-tracking during visual probe tasks (based on [16] but using pictures instead of words) [18, 19]. A similar study did not find such differences between hungry and satiated women or weight groups in a viewing task showing pictures of objects compared with pictures of high energy foods. In a dot probe task done in the same individuals however, overweight and obese individuals did automatically direct their attention to food-related stimuli to a greater extent than normal-weight individuals, in particular when hungry [5].

Although overweight children may have a higher food cue sensitivity than normal-weight children (as shown by their performance on a Stroop task [20] using food-related words [21]), it may be the case that all children have an attentional bias towards palatable foods when measured with an imbedded word test [22] and a visual probe task [23, 24]. When comparing children to adults, adults are initially strongly attracted by unhealthy foods, but they shift their attention from the unhealthy to the healthy foods, suggesting a self-regulation process of avoidance when measured in with naturalistic viewing paradigm [25]. Children, on the contrary, attend more strongly to unhealthy foods and do not shift their attention away [26]. In older adults and elderly, food cue sensitivity has not been studied. In summary, overweight individuals may be more sensitive to food cues, and hunger status likely plays a role in the differences in study outcomes. Children may have a bias towards (palatable) unhealthy food and find it hard to direct their attention away from it.

#### Self-Control Capacity

The ability to regulate behavior effectively is relevant in many aspects of daily life, such as the consumption of healthy food, purchase decisions, or sexual behavior. Self-control refers to the ability to withhold a response with an immediately rewarding outcome in favor of a response with an outcome that is more advantageous in the long run. Thus, self-control is an important part of healthy food choice, as a lack of it may result in unhealthy food choices and overconsumption. In line with this, self-control capacity has been shown to be negatively related with body weight [27, 28].

In adults, obesity is associated with impaired response inhibition capacity, greater delay discounting, and reduced executive function in general [28–30]. Response inhibition refers to suppression of actions that are inappropriate in a given context and that interfere with goal-driven behavior [31]. Response inhibition is most often measured with go/no-go and stop-signal tasks (e.g., [32, 33]). Delay discounting refers to the tendency for more remote outcomes to have less value [34]. This is often measured with a delay discounting task, in which a choice has to be made between an amount of money available immediately or a larger amount of money available later. In children, delay of gratification has been measured with tasks in which they are asked to resist a small reward (e.g., a marshmallow) for 15 min in favor of multiple or greater rewards later [35]. Executive functioning is an umbrella term that includes cognitive control, the ability to sustain or flexibly redirect attention, the inhibition of inappropriate behavioral responses, initiation and execution of strategies, and the ability to flexibly switch among strategies [36]. All of these constructs contribute to self-control ability. In children, self-control improves as they grow older. Accordingly, children and adolescents are more impulsive than adults, as is apparent from both response inhibition tasks and choice impulsivity tasks (such as delay of gratification and delay discounting) [37••]. However, the relative level of self-control at a given age is a stable personality trait. In accordance with findings in adults, there is a consistent relationship between self-control and weight status in children. Overweight and obese children and adolescents exhibit reduced executive function [38, 39], and less cognitive flexibility as measured with the Wisconsin Card Sorting Test (WCST) [40]. In line with this, a delay of gratification task at preschool age even predicts BMI 30 years later [41]. As adults age, self-control may improve as they become less impulsive and delay discounting tendencies decline [42]. To conclude, weight status and age are both related to self-control; and because of its stability over time and predictive value for weight status, further research on self-control mechanisms in food choice and how to increase self-regulatory success.

For a summary of the behavioral results see Table 1. In the field of food decision-making, older adults and elderly have not been the subject of many studies. Thus, it remains unclear which factors influence food choices later in life.

### Neural Correlates of Food Decision-Making

Food choices are made in the brain, integrating a multitude of neural and hormonal signals reflecting internal state and the environment [43]. The brain does not reach full maturity until 21 years of age. Furthermore, not all brain areas mature at the same rate; relatively greater changes have been reported in the prefrontal cortex (PFC) compared with the other brain regions between the age of 8 and the early 20s for synaptogenesis [44], gray matter reduction [45], myelination increase [46], and resting level metabolism [47]. Areas in the PFC, such as its lateral areas, mediate the capacity to voluntarily inhibit desire for a short-term reward in favor of a (larger) long-term reward [48] and are thus important for self-control. As people grow old, there are gradual structural changes such as decreases in gray matter density and synaptic pruning and cell shrinkage [49].

How the brain reacts to food is often measured by functional magnetic resonance imaging (fMRI). The most widely used fMRI technique is blood-oxygen level-dependent (BOLD) fMRI. This form of fMRI exploits the fact that at a site of increased neuronal firing (brain activation), changes in blood oxygenation occur which lead to a small increase in the fMRI signal (∼1 %). Neuroimaging studies that have examined processes underlying food decision-making can be divided into two categories: anticipation to food upon cue exposure and food choice. In Table 2, an overview is given of the brain regions most commonly implicated in food anticipation and choice.

**Table 2:** Brain areas most consistently implicated in studies on food anticipation and food choice

Area	Function
Ventromedial prefrontal cortex (vmPFC)/OFC	Incentive/subjective value of food
Dorsolateral prefrontal cortex (dlPFC)	Self-control, anticipation of reward, monitoring of behavioral consequences
Anterior cingulate cortex (ACC)	Conflict monitoring, self-control
Amygdala	Emotion, assigns value to sensory stimuli (valence)
Hippocampus	Episodic memory and learning aspects of food-related behaviors such as dietary learning
Striatum	Reward processing, motivated behaviors, and incentive learning
Insula	Interoception, encoding of multimodal sensory features of foods
Lateral occipital complex/occipital gyrus	Visual attention, object recognition
Primary motor cortex/precentral gyrus	Motor coordination and planning, motivation
Posterior parietal cortex	Subjective value, decision-making

#### Neural Correlates of Anticipation to Food

The process of food choice starts with the anticipation phase, when food or food-related cues are perceived or thought of. Upon perception of a food cue, multiple processes occur in the brain such as preparation for food ingestion and food evaluation [43, 50]. Examining brain responses to food cue exposure helps to elucidate the mechanisms underlying eating behavior. This is supported by studies showing that brain reactivity to food cues predicts things like future weight gain in adolescent girls [51] and women [52], food choice [53, 54], snack consumption [55], weight status [56], and outcome in a weight-loss program [57]. When normal-weight individuals look at food pictures compared with non-food pictures, areas in the appetitive brain network become active. This network centers around four interconnected brain regions: (1) the amygdala and hippocampus, (2) the orbitofrontal cortex (OFC) and ventromedial prefrontal cortex (vmPFC), (3) the striatum, and (4) the insula [50, 58]. Furthermore, brain areas involved in attention and visual processing (lateral occipital complex) are consistently more active in response to food compared with non-food pictures [50].

Functional neuroimaging has provided a means to investigate on a neural level whether overweight and obese individuals are more sensitive to food cues (see, e.g., Schachter’s externality hypothesis, which states that obese people are more reactive to external food cues and less sensitive to internal hunger and satiety signals than normal-weight individuals [59]) and may thus exhibit greater anticipatory brain activation upon food cue exposure. Indeed, overweight and obese individuals have increased activation in response to food cues in regions associated with cognitive evaluation of salient stimuli (OFC, dorsomedial prefrontal cortex; dmPFC, anterior cingulate cortex; ACC), motor responses (precentral gyrus) and explicit memory (parahippocampal gyrus), when compared with normal-weight individuals. Additionally, they have reduced activation in regions linked to cognitive control (dorsolateral prefrontal cortex; dlPFC) and interoceptive awareness (insular cortex) compared to normal-weight individuals [60•]. Furthermore, hunger state has a differential effect on obese than on normal-weight individuals. When hungry, obese individuals show greater activation in areas involved in emotion and memory (amygdala/hippocampus), and reduced activation in areas involved in interoception (insula) than those with normal-weight. When satiated, obese individuals have greater activation in reward areas (caudate body/striatum), areas associated with cognitive evaluation of salient stimuli (dmPFC), and attention (supramarginal gyrus) than normal-weight individuals [61]. Thus, overweight and obese individuals may have a stronger anticipatory response to food in areas involved in evaluation and memory and a lower response in areas important for cognitive control and interoception. Food-related brain responses of overweight and obese people may be differentially affected by satiation as they may have a higher reward response than normal-weight people when satiated. This may make them more likely to eat even when they are not hungry.

In response to food cues, children most consistently activate the same areas as adults do, which are part of the appetitive brain network [62•]. There are some indications that children may not activate areas important for cognitive control (ventrolateral prefrontal cortex; vlPFC), but there are not enough studies in children to properly establish this [62•]. Only a handful of studies have looked at the difference in brain activation in response to food cues between normal-weight and overweight children. When comparing overweight and obese with normal-weight children, the former show higher activation during food anticipation in areas involved in cognitive control (dlPFC, vlPFC), interoception (insula), and cognitive evaluation of salient stimuli (OFC, ACC) [51, 63–65]. Overweight and obese children deactivate areas involved in visual attention (the middle occipital and fusiform gyrus), memory (the hippocampus and parahippocampal gyrus), and reward (the caudate/striatum) compared with normal-weight children [63]. In summary, children may have less inhibitory activation during food anticipation. Few studies have been done in overweight children and results appear to contradict those in adults, as children with overweight have a higher response in areas involved in cognitive control and interoception when compared with normal-weight children while the opposite is found in adults. Intriguing as this finding may be, given the small number of studies and large age ranges of children studied (8–18 years), future studies should directly compare normal and overweight children and adults. So far, no studies have addressed the neural correlates of food anticipation in older adults or elderly.

#### Neural Correlates of Food Choice

To date, the neural correlates of food choice have been studied relatively little. Various tasks and designs have been used to investigate aspects of the brain processes behind food decisions. These studies mostly use single or dual food choice paradigms [53, 66–73], willingness to pay for different foods [74–77], or auction paradigms [78]. However, tasks, types of choices, stimuli, and participant characteristics vary greatly between studies. In the decision-making process, the different attributes of the stimuli (e.g., taste, healthiness, size, and packaging) are valued, weighed, and integrated into a single stimulus value [79, 80••]. Neuroimaging studies have consistently shown that this stimulus value is encoded in the vmPFC, both for food and non-food (e.g., monetary) items [70–77, 81]. For a comprehensive review on the neurocomputational perspective of dietary choice see Rangel [80••].

In the context of overconsumption, it is interesting to investigate how healthiness of food impacts the food choice process. To elucidate what happens in the brains of people motivated to make healthy choices, dieters can be examined. When dieters successfully make healthy choices, the value signal encoded in the vmPFC is increased by the healthiness of the choice option. During healthy choice, vmPFC activation is modulated by the dlPFC when self-control is necessary (e.g., when refusing an unhealthy, but tasty food) [72]. In dieters that do not successfully exercise self-control, the value signal in the vmPFC only reflects taste, while in successful self-controllers it incorporates both taste and health. Intriguingly, these neural mechanisms underlying successful self-control can be activated by merely asking people to consider the healthiness of the food. When considering healthiness, the vmPFC value signal incorporates the health aspects of the food even in individuals without an explicit health goal. Furthermore, the vmPFC signal is again modulated by the dlPFC, and they make healthier choices [73]. In everyday life, a health cue might come in the form of a health label used in marketing (such as “high in calories” or “low fat content”). When labels like this are shown alongside food in a food choice task, the healthiness of the foods is encoded in the amygdala (emotion) [66]. Interestingly, there is a negative coupling between amygdala and dlPFC when these health labels are shown [66]. The difference between the neural responses to health considerations and health labels may be caused by the fact that the health labels were shown more implicitly compared with the explicit instruction to consider healthiness. Alongside health labels, health information is commonly encountered in the shape of nutritional value tables on food packaging. However, a more graphic design, a traffic light system, has been proposed as an alternative and is more effective in promoting healthy choices [82]. When the neural responses to this traffic light label are compared with text-based nutritional information, red traffic light signaling (for unhealthy foods) activates the dlPFC, and there is increased coupling between dlPFC and vmPFC [83•].This suggests that explicitly asking to attend to healthiness or a graphic health label leads to different neural processing than implicitly showing a health label. This should however be further examined.

An interesting way to look at the effect of caloric content and tastiness of foods is to make choice-pairs based on liking. When people choose a high calorie product over a low calorie product, while they are sated and they have rated the foods as equally tasty, the superior temporal sulcus, a brain area involved in processing biological relevant information is activated [69]. This suggests that even when motivation to eat is low, the brain still tracks caloric value. Choice-pairs can furthermore be made challenging by design, by pairing a liked high calorie food with a less liked low calorie food. Weight-concerned women, who are trying to limit their energy intake but are generally unsuccessful in this, show lower activation in the anterior cingulate cortex, an area involved in valuation and conflict monitoring when making challenging choices, and accordingly fail to choose in line with their dieting goal [67].

To our knowledge, the effects of weight status or age on the neural correlates of food choice have not yet been examined. However, since the dlPFC is among the last brain regions to mature, the self-control system may be underdeveloped in children, which would make healthy food decisions more challenging for them. Furthermore, lower dlPFC activation in overweight/obese adults during food anticipation suggests that they may have poorer self-control.

In conclusion, there is a growing body of work on the neural correlates of food choice. Valuation activity in the vmPFC appears to be mostly related to tastiness in normal-weight individuals. When considering the healthiness of the food, or attending to graphic health labels, health value is encoded in the dlPFC and positively modulates vmPFC activation. More implicit health information is encoded in the amygdala and negatively coupled with dlPFC activation. Even when satiated, the brain tracks caloric content during choice, and the lack of conflict-related brain activation may cause self-control to fail in weight-concerned women. Future studies should expand this by exploring the role of weight status and age on healthy decision-making.

## Discussion

Although the obesity epidemic has caused increased attention for food decision-making, there are still several underexplored areas. Without longitudinal studies, it is impossible to establish the causality of any of the factors discussed that influence food decision-making. For example, we cannot say whether poor self-control causes weight gain or that the state of being obese causes diminished self-control. Large population-based cohorts can hopefully be used to collect valuable information on how weight gain and weight loss impact food decision-making. Furthermore, there is an overrepresentation of college-aged adults in the literature, little work has been done in children, and almost no work has been done in older adults and elderly, while the latter two are very important groups to target. Since an overweight child has a large chance to develop into an overweight adult, prevention of overconsumption of unhealthy foods and formation of healthy eating habits in children is crucial. Moreover, many Western countries have an increasing elderly population, and many health problems experienced by the elderly such as type 2 diabetes, cognitive decline, and cardiovascular disease have been associated with overweight/obesity and specific dietary factors, such as saturated fat intake and vitamin E and B12 deficiency [84]. Thus, additional research into food choice in older adults could be beneficial for multiple health outcomes. Lastly, the field would greatly benefit from standardization of methods, both in behavioral and neuroscience studies, to decrease between study variability and foster meta-analyses and replication studies.

## Conclusions

Age and weight status both significantly influence the food decision-making process; however, more work, especially in children and elderly, is needed to better understand the drivers of dietary decision-making. Behavioral studies show that in particular poor self-control may have an adverse effect on food choice in children and in those with overweight and obesity. Neuroimaging studies show that overweight and obese individuals have different neural responses to food in the brain regions involved in reward, self-control, and interoception. More research into the neural correlates of food choice may provide better insight in the effects of age and weight on the food decision-making process and provide targets for healthy eating interventions, which may be tuned to different subgroups like children or dieters. Longitudinal studies including individuals differing in weight status will be invaluable to unravel the causal factors that shape food decisions.
